# Enhancement of anti-tumor effects of 5-fluorouracil on hepatocellular carcinoma by low-intensity ultrasound

**DOI:** 10.1186/s13046-016-0349-4

**Published:** 2016-04-22

**Authors:** Zheng Hu, Guixiang Lv, Yongning Li, Enze Li, Haixia Li, Qi Zhou, Bin Yang, Wenwu Cao

**Affiliations:** Laboratory of Sono- and Photo-theranostic Technologies, Harbin Institute of Technology, Harbin, 150080 China; Department of Biochemistry and Molecular Biology, Harbin Medical University, Harbin, 150086 China; Department of Mathematics, and Materials Research Institute, The Pennsylvania State University, University Park, 16802 PA USA

**Keywords:** Low-intensity ultrasound, 5-fluorouracil, Anti-tumor effects, Hepatocellular carcinoma, Reactive oxygen species

## Abstract

**Background:**

Hepatocellular carcinoma (HCC) accounts for 75 % of liver cancers and is the second most lethal cancer, associated with its multiple etiologies, poor prognosis and resistance to chemotherapy drugs. Chemotherapy treatment on HCC suffers low efficacy of drug uptake and can produce a range of side effects. Here we report an investigation on the effect of a combined treatment on human hepatocellular carcinoma BEL-7402 cells using low-intensity ultrasound (US) and 5-fluorouracil (5-FU).

**Methods:**

The uptake of 5-FU was measured by the high-performance liquid chromatography (HPLC). DNA damage was detected by the comet assay. MTT assay was used to examine cell viability. Intracellular reactive oxygen species (ROS) and mitochondrial membrane potential (Δψm) were respectively detected by the fluorescent probes DCFH-DA or JC-1. Endogenous apoptosis-associated proteins were analyzed by the western blot and immunohistochemistry. Histopathological changes were evaluated by the hematoxylin and eosin (H&E) staining. Cell apoptosis was evaluated by the TUNEL and flow cytometry assays. Cell proliferation was measured using the immunohistochemical staining of PCNA.

**Results:**

Our results showed that low-intensity US (1.1 MHz, 1.0 W/cm^2^, 10 % duty cycle) significantly enhanced the uptake of 5-FU, 5-FU-mediated DNA damage and reactive oxygen species (ROS) generation. The increased ROS production up-regulated the p53 protein level, which led to the up-regulation of Bax and down-regulation of Bcl-2. The enhancement of ROS generation and the activation of the apoptosis-associated proteins further triggered the collapse of mitochondrial membrane potential, released cytochrome c from mitochondria into cytosol and activated the mitochondria-caspase pathway, and cell apoptosis. Such enhanced effects could be partially blocked by the ROS scavenger N-acetylcysteine (NAC). Overall, low-intensity US combined with 5-FU led to an effective inhibition of tumor growth and prolonged overall survival of BEL-7402 HCC-bearing nude mice by more than 15 % compared with 5-FU treatment alone.

**Conclusions:**

Our results showed that low-intensity ultrasound combined with 5-FU produced much enhanced synergistic anti-tumor effects via enhanced ROS production in treating HCC.

**Electronic supplementary material:**

The online version of this article (doi:10.1186/s13046-016-0349-4) contains supplementary material, which is available to authorized users.

## Background

Hepatocellular carcinoma (HCC) is the sixth most prevalent cancer and the second most lethal cancer, with the rate of 5-year survival below 20 % [1]. Statistics show that there were nearly 800,000 new cases worldwide in 2012 and the number increases each year [2]. The etiology and pathogenesis of HCC are not yet fully elucidated because of its heterogeneity and multiple causes, but chemotherapy is a treatment modality for HCC but the effect is not satisfactory [3]. The 5-fluorouracil (5-FU), as an important antimetabolite and cell cycle-targeting drug, has been widely used for the treatment of gastric, colorectal, and breast cancers [4], and it exerts its anti-tumor effects through the inhibition of RNA/DNA processing and thymidylate synthase [4]. In recent years, 5-FU proved to be beneficial in treating advanced HCC in the FOLFOX4 regimen (including infusional 5-FU, leucovorin, and oxaliplatin), which increased the overall survival of HCC patients by 1.47 months [5]. However, the low efficacy of drug uptake compels doctors to use higher doses of 5-FU, causing a range of side effects, including leukopenia, nausea, emesis, and skin reactions. Therefore, there is an urgent need to find better ways that can improve the sensitivity of HCC to chemotherapy, and to reduce drug-dosage so as to reduce side effects.

Therapeutic ultrasound (US), especially low-intensity US, has gained increasing attention in recent years [6]. There are several important advantages of US assisted therapies, particularly sonodynamic therapy (SDT), in which ultrasonic waves can penetrate deep into the tissue to stimulate sonosensitizers that are specifically localized in the tumor to achieve localized anticancer effects [7]. In general, US can cause both physical effects (such as heat, cavitation, and mechanical force) and biochemical effects (such as reactive oxygen species) to effect tumor cell damage and apoptosis [8]. Some investigations demonstrated that low-intensity US enhanced anti-tumor effects of chemotherapeutic agents, including doxorubicin [9], cyclophosphamide [10], docetaxel [11], cisplatin [12], etc. We also demonstrated the efficacy of combined chemotherapy on human tongue cancer cells in vitro and in vivo using *scutellarin* and US, and found that the combined treatment with much reduced drug dosage significantly inhibited tumor angiogenesis, suppressed cancer cell proliferation and induced cancer cell apoptosis [13].

The aim of this work is to determine whether low-intensity US can promote the uptake of 5-FU by HCC cancer cells, and to see if low-intensity US combined with 5-FU (US + 5-FU) can effectively inhibit the growth of BEL-7402 HCC-bearing nude mice. We will look at the changes in the 5-FU-mediated ROS generation and to find out if the cancer cell apoptosis is via the mitochondria-caspase pathway.

## Methods

### Chemicals and antibodies

The 3-(4,5-Dimethyl-2-thiazolyl)-2,5-diphenyl-2H-tetrazolium bromide (MTT; 98 % purity), 2-(4-Amidinophenyl)-6-indolecarbamidine dihydrochloride (DAPI; purity by HPLC: ≥ 98 %), 5-FU (purity by HPLC: ≥ 99 %), and N-acetylcysteine (NAC; purity by TLC: ≥ 99 %) were purchased from Sigma-Aldrich (St Louis, MO, USA). Mito-Tracker Green and Z-VAD-FMK were purchased from Beyotime (Shanghai, China). Mouse anti-PCNA (60097) was purchased from Proteintech (Chicago, IL, USA). Mouse anti-p53 (ab26) and the rabbit anti-cytochrome c (ab133504) were purchased from Abcam (Cambridge, MA, USA). Rabbit anti-Bcl-2 (sc-492), rabbit anti-Bax (sc-526) and mouse anti-β-actin (sc-47778) were purchased from Santa Cruz Biotechnology (Santa Cruz, CA, USA). Rabbit anti-cleaved caspase-3 (9661) was obtained from Cell Signaling Technology (Beverly, MA, USA).

### Cell culture

Human hepatocellular carcinoma BEL-7402, Hep G2, and HuH-7 cell lines were provided by China Center for Type Culture Collection (Wuhan, Hubei, China). Cells were incubated in Dulbecco′s modified Eagle′s medium (DMEM) supplemented with 10 % fetal bovine serum at 37 °C in an atmosphere of 95 air and 5 % CO_2_.

### Ultrasound device and intensity measurement

**Fig. 1:**
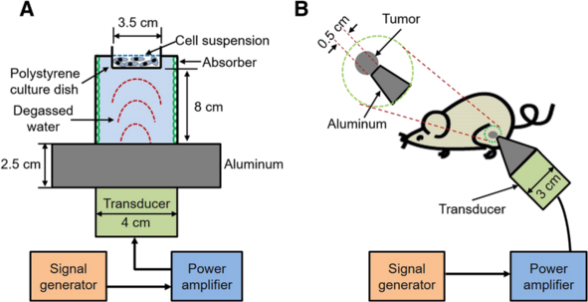
Schematic diagram of low-intensity ultrasound device and the experimental setup. The tone-burst ultrasonic transducer (1.1 MHz center frequency, 10 % duty factor, 100 Hz pulse repetition rate) for the in vitro (**a**) and in vivo (**b**) experiments

For the in vitro experiments (Fig. 1a), a 3.5 cm diameter polystyrene cell culture dish (Corning, NY, USA) with a tumor cell suspension was placed in degassed water 8 cm above the 4.0 cm diameter ultrasonic transducer (nonfocused, 1.1 MHz center frequency, 10 % duty cycle, 100 Hz pulse repetition frequency) with a 2.5 cm thick aluminum transmission medium. An ultrasonic wave absorber was fitted to the internal surface of the acrylic sonication tank to avoid wave reflections. The US intensity was measured using an HNC-1000 needle-type hydrophone (0.1 cm active element size, 1–20 MHz bandwidth) (Onda, Sunnyvale, USA). For the in vivo experiments (Fig. 1b), a 3.0 cm diameter nonfocused ultrasonic transducer was attached to a tapered aluminum buffer head with a 0.5 cm diameter front surface, which was directly in contact with tumor site skin using acoustic couplant gel. A water-proof thermocouple was immersed in the solution for measuring the temperature during the in vitro experiments. For the in vivo experiments, the temperature measurements were on the skin above the tumor under the ultrasound transducer. Due to the low intensity level, the temperature increase in all experiments was less than 2 °C.

### High-performance liquid chromatography (HPLC) and measurement of drug uptake

The uptake of 5-FU was measured using an Agilent 1200 series HPLC system (Santa Clara, CA, USA). The chromatographic conditions were as follows: the chromatographic column was a 20RBAX Eclipse XDB-C18 column (4.6 × 150 mm, 5 μm particle size); the mobile phase was 10 mM phosphate-buffered saline (PBS; 137 mM NaCl, 2.7 mM KCl, 10 mM Na_2_HPO_4_, and 2 mM KH_2_PO_4_, pH 7.4); the flow rate was 1.0 ml/min; the column temperature was 25 °∁; the detection wavelength was at 265 nm and the injection volume was 10 μl. Briefly, cell suspensions (1 × 10^6^) with 10 μg/ml 5-FU were immediately sonicated and incubated for 30 min. The cells were disrupted and harvested in cold PBS. The supernatant was injected in the column for analysis.

### Intracellular ROS detection

The detection was performed by using a Reactive Oxygen Species Assay Kit (Applygen, Beijing, China). Briefly, cells pretreated with DCFH-DA (10 μM) at 37 °C for 10 min were sonicated and examined using an Olympus IX71 invert fluorescence microscope (Tokyo, Japan) after 20 min of incubation. The ROS production was calculated using the IPP software.

### Comet assay

DNA damage was detected using the alkaline comet assay 24 h after the different treatments as described by He et al. [14]. The cells were stained with PI, and the tail moment (% Tail DNA) was evaluated using a fluorescence microscope. The percentage of comets formed reflected the degree of DNA damage.

### Cell viability and treatment protocol

Cell viability was evaluated by the MTT assay. Cells were treated with the following dosages of 5-FU: 0, 2, 5, 10, 20, 40, 60, 80, and 100 μg/ml. After 48 h of incubation, cells were treated with MTT (final concentration of 0.5 mg/ml) for 4 h and shocked with 150 μl DMSO per well for 10 min. The absorbance value was measured at 490 nm using SpectraMax M3 plate reader (Molecular Devices, Sunnyvale, CA, USA). For the in vitro US + 5-FU treatment, cells were divided into four groups: (1) Control; (2) US; (3) 5-FU; (4) US + 5-FU. After 30 min of incubation, cell suspensions with 10 μg/ml 5-FU were immediately sonicated by US (1.1 MHz, 1 W/cm^2^, 10 % duty cycle) for 5 min and drug residues were removed using saline. After 48 h of incubation, cell viability was measured and protein expression was analyzed by western blot. In addition, some cells were also pretreated with NAC (5 mM) or z-VAD-FMK (10 μM) for 1 h prior to loading 5-FU to study the ROS effects.

### Mitochondrial membrane potential detection

The detection was carried out by using a Mitochondrial Membrane Potential Detection Kit (Beyotime, Shanghai, China). Immediately after the treatments, cells were incubated with JC-1 for 20 min and examined by an inverted fluorescence microscope. The fluorescence intensities were calculated using the IPP software.

### Immunofluorescence

Five hours after the treatments, cells were incubated with 200 nM Mito-Tracker Green for 30 min. Then the cells were blocked with 1 % BSA (dissolved in PBS and 0.05 % Tween-20) for 20 min and incubated with cytochrome c antibody (1:100) overnight at 4 °C. Alexa Fluor 555 secondary antibody (1: 500; Beyotime, Shanghai, China) was added for 1 h followed by the addition of DAPI for 15 min. Finally, cells were examined using an Olympus BX51 fluorescence microscope (Tokyo, Japan). Fluorescence images of cytochrome c, Mito-Tracker Green, and DAPI were taken at the excitation of 555 nm, 490 nm and 364 nm, respectively.

### Immunohistochemistry

As described in a previous work [15], the tissue sections were dewaxed, rehydrated, and retrieved in 10 mM citrate buffer pH 6.0 (10 mM citrate buffer and 0.05 % Tween-20). Then these sections were immersed in 3 % hydrogen peroxide for 15 min and stained with different primary antibodies (1:100) with the non-immunized goat serum at 4 °C overnight. After incubating with secondary antibodies (1:3000) for 30 min, the sections were stained with DAB (Zhongshan Goldenbridge, Beijing, China). Immunopositive cells were randomly selected from 10 fields (magnification × 200) and calculated using the IPP software.

### Western blot

Cell lysates were prepared in RIPA buffer {50 mM Tris–HCl pH 7.5, 150 mM NaCl, 1 mM EDTA, 1 % (v/v) Triton X-100, 1 % (w/v) sodium deoxycholate, and 0.1 % (w/v) SDS} in the presence of a proteinase inhibitor cocktail Complete mini (Roche, Indianapolis, IN, USA), separated by 12 % SDS-PAGE, and transferred to nitrocellulose membranes. After blocking using 5 % non-fat dried milk with TBST buffer (10 mM Tris–HCl pH 8.0, 150 mM NaCl, and 0.05 % Tween-20), membranes were incubated with primary antibodies-p53 (1:1000), Bcl-2 (1:500), Bax (1:500) and cleaved caspase-3 (1:1000) overnight at 4 °C and then incubated with horseradish peroxidase-conjugated secondary antibodies (1:5000) for 1 h. Bands were detected using a Pro-light HRP Chemiluminescence Kit (TIANGEN, Beijing, China).

### Flow cytometry

Apoptosis evaluation was performed by using a FITC Annexin V Apoptosis Detection Kit I (BD Biosciences, San Diego, CA, USA). Briefly, 5 h after the treatments, cells were suspended in 500 μl binding buffer (0.01 M Hepes/NaOH pH 7.4, 140 mM NaCl, 2.5 mM CaCl_2_) with a mixture of 5 μl FITC Annexin V (1 μg/ml) and 5 μl PI (2 μg/ml) to incubate for 15 min. The samples (20,000 events) were analyzed using a BD FACS Canto II flow cytometer (San Diego, CA, USA).

### Tumor treatment protocol

Six- or 8-week-old female BALB/c nude mice (about 20 g/mouse) were purchased from the SLAC Laboratory Animal Company (Shanghai, China). BEL-7402 cells (1 × 10^7^) with 200 μl DMEM were subcutaneously injected into the flanks of nude mice to make cancer models. When the tumors reached ~100 mm^3^, the mice were randomly divided into 12 groups with 6 mice per group for three different experiments: (1) Screening for suitable dosages of 5-FU. The four groups were as follows: Control, untreated control mice; 5-FU, mice received chemotherapy treatment using 5, 10, 20 mg/kg 5-FU, respectively, by intraperitoneal injection; (2) Evaluation of the anti-tumor efficacy. The four groups were as follows: Control, untreated control mice; US, mice treated with US (1.1 MHz, 1 W/cm^2^, 10 % duty cycle) alone for 10 min; 5-FU, mice received 10 mg/kg 5-FU; US + 5-FU, mice treated with US after 30 min of 5-FU administration. The mice were euthanized after 17 days; (3) Survival analysis. The last four groups were divided in the same way as in (2). The mice were euthanized when they lost 10 % of body weight to avoid artifacts. Tumor volume and body weight were measured every 2 days. Tumor diameter was measured using a vernier caliper and tumor volume was calculated by the formula *V* = π/6 × *L* × *S*^2^ (here *L* and *S* are the long and short diameters, respectively). All experiments were performed according to the standards developed by the Experimental Animal Care and Use Committee of Harbin Institute of Technology.

### Hematoxylin and eosin (H&E) staining

Tumors were fixed using 10 % formalin for at least 24 h. Samples were then paraffin-embedded, sectioned, and stained. Histopathological changes were observed using a light microscope.

### TUNEL assay

Tumor xenografts were detected by using an *In Situ* Cell Death Detection Kit (Roche, Mannheim, Germany) as described in a previous work [16]. The apoptosis index (AI) was evaluated by counting the number of TUNEL-positive (brown-stained) cells in 10 randomly selected fields (magnification × 200) and calculated the integrated optical density (IOD) values by using an Image-Pro Plus (IPP) software.

### Statistical analysis

Survival analyses were estimated by using a SPSS software (IBM SPSS, Armonk, NY, USA) using the Kaplan-Meier method. Data was compared with the log-rank test and expressed as the means ± SD. Statistical differences were evaluated by using a one-way ANOVA Dunnett’s test. Differences between any two groups were assessed by the Student Newman-Keuls test, with *p* < 0.05 considered to be statistically significant.

## Results

### Low-intensity ultrasound enhanced the uptake of 5-FU and 5-FU-mediated ROS generation

**Fig. 2:**
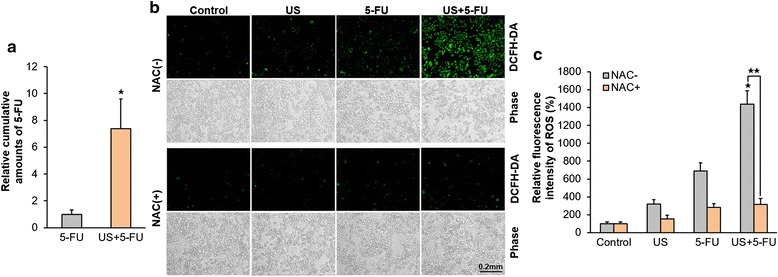
The uptake of 5-FU and 5-FU-mediated ROS production after the application of ultrasound. **a** Relative cumulative amounts of 5-FU in the 5-FU alone and US + 5-FU groups in BEL-7402 cells (^★^ *p* < 0.05 *vs.* 5-FU group). **b** Representative intracellular ROS (green fluorescence) after the treatment. **c** The relative level of intracellular ROS by the fluorescent intensity quantified with IOD values. Data was presented as the mean ± SD (*n* = 6, ^★^ *p* < 0.05 *vs.* other three groups; ^★★^ *p* < 0.05 NAC-treated *vs.* NAC-untreated US + 5-FU group)

To investigate the underlying mechanism of US + 5-FU-mediated anti-tumor effects, we measured intracellular 5-FU uptake in vitro. As shown in Fig. 2a, after the application of ultrasound, the cumulative amounts of 5-FU in the US + 5-FU group (9.3 × 10^−7^ μg per cell) increased about 7-fold compared with the 5-FU alone group (1.3 × 10^−7^ μg per cell; *p* < 0.05), indicating that low-intensity US effectively increased the uptake of 5-FU in BEL-7402 cells.

Previous studies have verified that intracellular ROS plays a key role in 5-FU-mediated anti-tumor effects [17, 18]. Here we monitored intracellular ROS levels and found that compared with the Control group (100 ± 21.1 %), the relative green fluorescence intensities of ROS increased 3-fold in the US alone group (320.6 ± 50.2 %) and 7-fold in the 5-FU alone group (689.8 ± 90.2 %; Fig. 2b, c). However, the fluorescence intensities of the US + 5-FU treatment increased significantly by as much as 14-fold (1437.4 ± 150.7 %; *p* < 0.05 *vs.* other three NAC-untreated groups), and these fluorescence intensities were reduced by as much as 78.0 ± 10.3 % by the ROS scavenger NAC (316.5 ± 66.2 %; *p* < 0.05 *vs.* NAC-untreated US + 5-FU group), indicating that US + 5-FU could effectively enhance ROS generation. This increased ROS generation was not due to the sonochemical effect because the 5-FU was not a sonosensitizer, but due to the much increased 5-FU uptake with the help of low intensity ultrasound. The ROS was primarily generated inside the cells.

### Low-intensity ultrasound combined with 5-FU induced DNA damage and ROS-mediated cell death

**Fig. 3:**
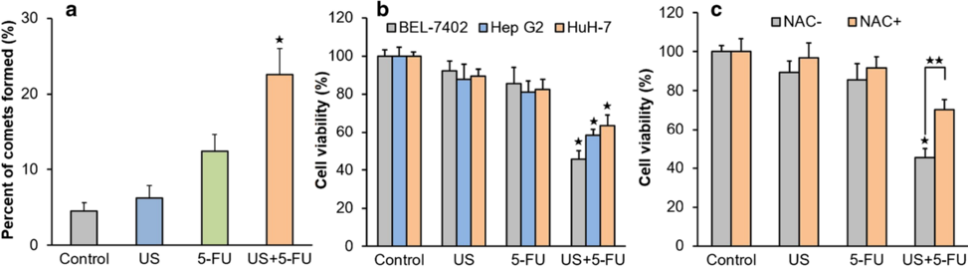
The DNA damage and cell viability after the treatment of low-intensity ultrasound combined with 5-FU. **a** DNA damage was evaluated by the tail moment. **b** Cell viability in the BEL-7402, Hep G2, and HuH-7. **c** Cell viability in the Control, US, 5-FU, and US + 5-FU groups with and without NAC. Data was presented as the mean ± SD (*n* = 6, ^★^ *p* < 0.05 *vs.* other three groups; ^★★^ *p* < 0.05 NAC-treated *vs.* NAC-untreated US + 5-FU group)

The results of the comet assays showed that 5-FU alone (12.4 ± 2.2 %) could induce DNA damage at 24 h compared with the Control and US groups (4.5 ± 1.3 and 6.2 ± 1.7 %, respectively; Fig. 3a). But more comets were detected in US + 5-FU group (22.6 ± 3.4 %; *p* < 0.05), indicating that low-intensity ultrasound combined with 5-FU synergistically increased the DNA damage.

To investigate the anti-tumor efficacy of US + 5-FU in vitro, we determined the suitable dosage of 5-FU. After 48 h of 5-FU administration, there was a dosage-dependent decrease in BEL-7402 cells (Additional file 1: Figure S1a). The 5-FU dose of 10 μg/ml was recommended for further experiments because it has a slight anticancer effect but not good enough. As shown in Fig. 3b, US + 5-FU significantly inhibited the growth of BEL-7402, Hep G2, and HuH-7 at 48 h after the treatment (with the inhibition ratios of 54.5 ± 4.6, 41.8 ± 3.3, and 36.5 ± 5.6 %, respectively; *p* < 0.05 *vs.* other three groups), whereas the effects of US or 5-FU alone only produced the slight inhibition ratios of 7.6 ± 4.8 and 14.5 ± 8.3 % in BEL-7402, 12.3 ± 7.8 and 18.8 ± 5.9 % in Hep G2, 10.6 ± 3.8 and 17.4 ± 5.3 % in HuH-7.

To see if the increased ROS accounts for the significantly enhanced anticancer effects after the US + 5-FU treatment, we further investigated the influence of ROS on BEL-7402 cell viability. As shown in Fig. 3c, when treated with the ROS scavenger NAC, cell viability in US + 5-FU group was indeed partly rescued (an increase of 24.7 ± 5.3 %; *p* < 0.05) compared with the group not treated by the NAC. These data suggested that US + 5-FU treatment exhibited anti-tumor effects by increasing 5-FU uptake and 5-FU-mediated ROS generation.

### Low-intensity ultrasound combined with 5-FU triggered the collapse of mitochondrial membrane potential via ROS

**Fig. 4:**
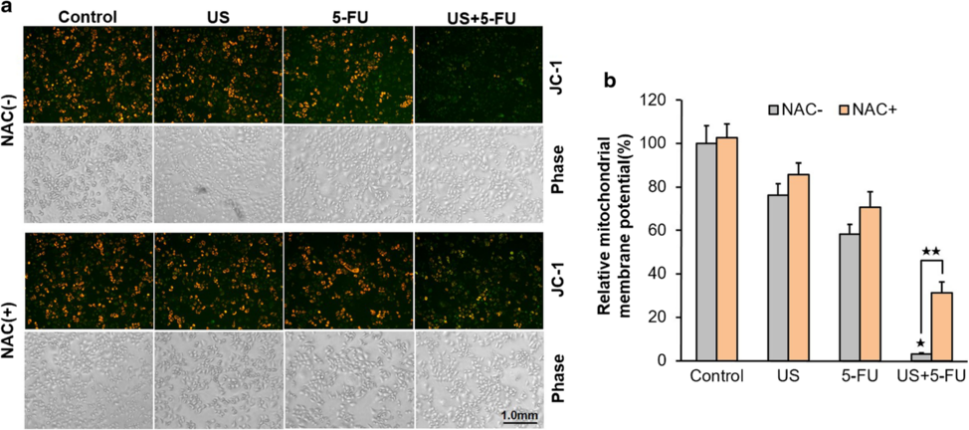
Alteration of mitochondrial membrane potential by the treatment of low-intensity ultrasound combined with 5-FU. **a** Representative mitochondrial membrane potential (Δψm) in BEL-7402 cells after the treatment. **b** The relative level of Δψm by the fluorescent intensity quantified with IOD values. Data was presented as the mean ± SD (*n* = 6, ^★^ *p* < 0.05 *vs.* other three groups; ^★★^ *p* < 0.05 NAC-treated *vs.* NAC-untreated US + 5-FU group)

To investigate the relationship among US + 5-FU, ROS and mitochondria damage, we examined potential changes of mitochondrial membrane potential (Δψm) in BEL-7402 cells. As shown in Fig. 4a, b, compared with the Control group, the relative Δψm in the US or 5-FU alone groups was decreased by 25–30 %, while that of the US + 5-FU group was further decreased by as much as 96.9 ± 13.5 % (*p* < 0.05 *vs.* other three NAC-untreated groups). When treated with NAC, the relative Δψm in the US + 5-FU group was increased by 10-fold compared with the NAC-untreated one (*p* < 0.05). These results suggested that the US + 5-FU-mediated ROS treatment was mainly responsible for the collapse of mitochondrial membrane potential.

### Low-intensity ultrasound combined with 5-FU activated mitochondria-caspase pathway via ROS

**Fig. 5:**
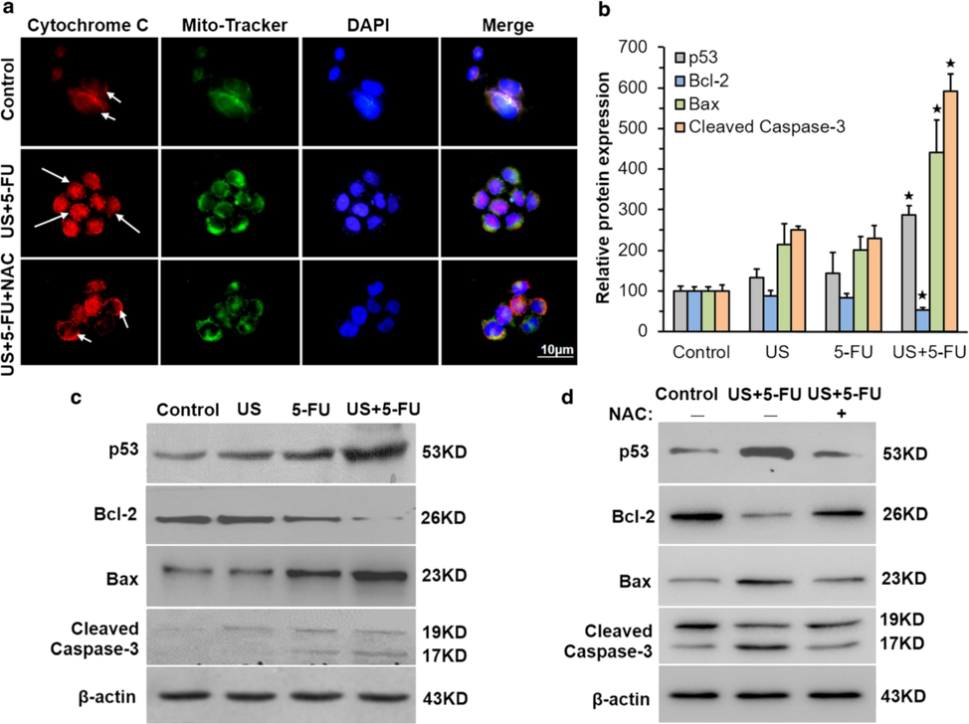
Activation of mitochondrial-caspase pathway of apoptosis by low-intensity ultrasound combined with 5-FU. **a** Representative fluorescence images of the translocation of cytochrome c in BEL-7402 cells. Cytochrome c in NAC-untreated US + 5-FU group diffused in the cytosol (long arrow), which was anchored to the mitochondria in the Control and NAC-treated US + 5-FU groups (short arrow). Representative expression of apoptosis-related proteins in vivo (**b**) and in vitro (**c**) after the treatment. **d** Representative expression of apoptosis-related proteins with and without NAC in BEL-7402 cells. Data was presented as the mean ± SD (*n* = 6, ^★^ *p* < 0.05 *vs.* other three groups)

To further examine how ROS was involved in US + 5-FU-mediated cell apoptosis, we evaluated the influence of both ROS and apoptosis-related proteins on the mitochondria-caspase pathway in BEL-7402 cells. As shown in Fig. 5a, a diffused red fluorescence was observed indicating the translocation of cytochrome c from mitochondria to the cytosol after the US + 5-FU treatment (long arrow), but there were no obvious changes in the Control and NAC-treated US +5-FU groups (short arrow). These data indicated that ROS was involved in the release of cytochrome c.

We also evaluated the change of apoptosis-related proteins both in vitro and in vivo. The immunochemical staining results in vivo (Fig. 5b and Additional file 2: Figure S2a-d) revealed that the levels of p53, Bax and cleaved caspase-3 proteins in the US + 5-FU group were much higher compared with that of the Control group (286.3 ± 15.3, 440.2 ± 31.8, and 591.4 ± 42.2 %, respectively; *p* < 0.05 *vs.* other three groups), whereas the levels of Bcl-2 were lower (52.7 ± 9.2 %; *p* < 0.05 *vs.* the other three groups). The alterations of p53, Bax, cleaved caspase-3 and Bcl-2 protein expression was also observed in the US + 5-FU group of BEL-7402 cells (Fig. 5c). When treated with NAC, the expression of these proteins was recovered (Fig. 5d), indicating that the combined US + 5-FU treatment indeed regulated the expressions of apoptosis-related proteins via ROS.

**Fig. 6:**
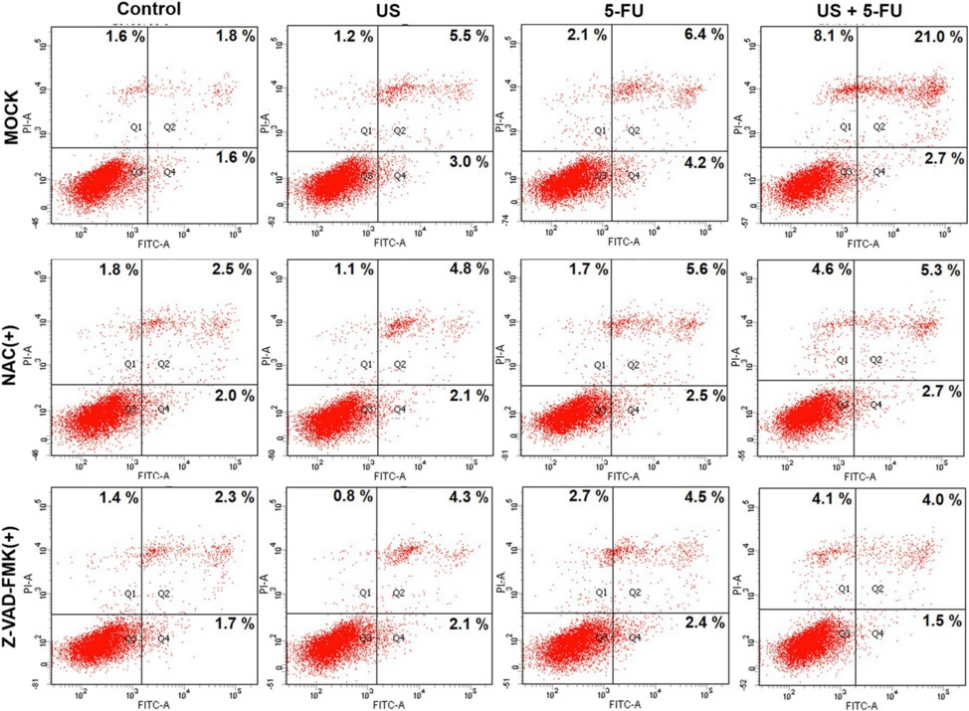
Induction of the caspase-dependent apoptosis by low-intensity ultrasound combined with 5-FU. Effects of NAC and Z-VAD-FMK on BEL-7402 cell apoptosis were determined by the Annexin V-PI assay

We further investigated the role of the US + 5-FU treatment in cell apoptosis by treating BEL-7402 cells with the ROS scavenger NAC or pan-caspase inhibitor Z-VAD-FMK*.* As shown in Fig. 6, US + 5-FU exhibited 23.7 % apoptosis index, which was significantly higher compared with the Control (3.4 %), US (8.5 %), and 5-FU groups (10.6 %), while the NAC or Z-VAD-FMK showed the potential to significantly reduce the apoptosis induced by the US + 5-FU treatment to 8.0 and 6.5 %, respectively. Meanwhile, the ratios of necrotic cells in the NAC-treated (4.6 %) or Z-VAD-FMK-treated group (4.1 %) were also reduced compared with the 8.1 % of the US + 5-FU group. These data demonstrated that the US + 5-FU treatment indeed induced caspase-dependent apoptosis via ROS.

### Low-intensity ultrasound combined with 5-FU exhibited significant anti-tumor effects

**Fig. 7:**
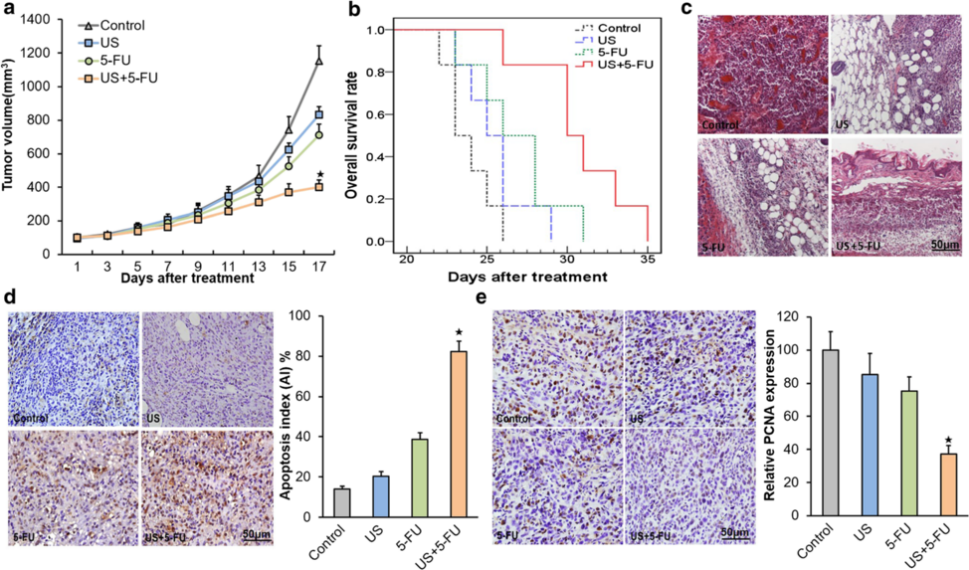
The anti-tumor effects on HCC after the treatment of low-intensity ultrasound combined with 5-FU. **a** Tumor-bearing nude mice in the Control, US alone, 5-FU alone, and US + 5-FU groups. **b** Overall survival analysis results by the Kaplan-Meier survival curves (log-rank test, *p* = 0.001). **c** Representative histopathological changes in the Control, US alone, 5-FU alone, and US + 5-FU groups. Representative TUNEL-positive (**d**) and PCNA-positive (**e**) cells from mice of different treatment groups. The apoptotic index (AI) and relative cell proliferation were calculated as the number of positive cells for each group. Data was presented as the mean ± SD (*n* = 6, ^★^ *p* < 0.05 *vs.* other three groups)

We evaluated the suitable dosage of 5-FU for the in vivo mice experiments. The administration of 5-FU resulted in some anti-tumor effects but not an effective retardation of tumor growth compared with the untreated control mice (*p* < 0.05; Additional file 1: Figure S1b). The 5-FU dose of 10 mg/kg was selected for further experiments. Treatment with US or 5-FU alone led to some degree of anti-tumor effect (with the inhibition ratios of 27.7 ± 4.1 and 38.2 ± 5.5 %, respectively) after 17 days (Fig. 7a), but for US + 5-FU treatment, a significant inhibition of tumor growth (inhibition ratio of 65.3 ± 3.8 %; *p* < 0.05) was observed compared with the Control group.

We further evaluated the overall survival of BEL-7402 HCC-bearing nude mice. The results were given in a Kaplan-Meier graph (Fig. 7b). The US + 5-FU group mediated a prolonged median survival of 30 days compared to the 5-FU group (26 days), US group (25 days), and Control group (23 days only). More detailed data are given in Table 1. The log-rank test revealed a 30 % increase in median survival after the US + 5-FU treatment compared with the Control group (*p* = 0.001) and a 15 % increase compared with the 5-FU alone treated group (*p* = 0.030). These data suggested that US + 5-FU indeed induced significant anti-HCC effects.

**Table 1:** Detailed results of the survival experiment in the BEL-7402 model

Treatment group	Maximum survival (days)	Minimum survival (days)	IST_median_ (%)^a^	Mean survival (days)	*p*-value^a^
Control (*n* = 6)	26	23	–	23.8 ± 0.6	–
US (*n* = 6)	29	24	8.7	25.5 ± 0.9	0.141
5-FU (*n* = 6)	31	24	13.0	26.8 ± 1.1	0.030
US + 5-FU (*n* = 6)	35	26	29.8	30.8 ± 1.3	0.001

IST = Improvement in survival compared with the untreated control group

^a^Compared to the untreated control group. Mean survival = means ± SD

In addition, H&E staining revealed a reduction in hyperemia, necrosis and the number of small vessels decreased in the US + 5-FU group compared with other three groups (Fig. 7c). Then we further assessed cell apoptosis and proliferation after the US + 5-FU treatment. The data in Fig. 7d showed that US + 5-FU exhibited a significant difference in apoptosis index (AI), i.e., 82.4 ± 5.2 % (*p* < 0.05) compared with the Control group, while treatment with US or 5-FU alone induced less than half of the apoptosis level. We also found that the PCNA staining intensities had significantly decreased in the US + 5-FU group (37.2 ± 5.3 %; *p* < 0.05 *vs.* other three groups), but there was much higher intensities of cell staining in the Control, US, and 5-FU groups (Fig. 7e).

## Discussion

Hepatocellular carcinoma is one of the most invasive and lethal tumors that responds poorly to chemotherapy. Therefore, there is an urgent need to find effective clinical treatments for HCC. In this work, we designed and constructed a low-intensity US device, aiming to explore the efficacy and mechanisms responsible for the enhancement of combined US + 5-FU treatment for HCC.

It was reported that US can promote the delivery of anticancer drugs into tumors [7, 19]. There were at least three possible mechanisms by which US could increase the permeability of the plasma membrane: heating effect, cavitation effect, and direct mechanical force [20]. The heat and cavitation effects were generally considered responsible for the US bio-effects [21]. However, in our experiment, we observed only a negligible temperature rise after the application of low-intensity US (1.1 MHz, 1 W/cm^2^, 10 % duty cycle), indicating that the heat effect was not the key mechanism here, consistent with the report from Chumakova et al. [22]. Our previous study had demonstrated that the main mechanism of low-intensity US combined with chemotherapy drug *scutellarin* was mechanical effect, which allowed drugs to be delivered to cancer cells by increasing the frequency and contact area between the drug and cells [13]. Herein, we found that low-intensity US could temporarily “open” cell membranes (Additional file 3: Figure S3) [23] and significantly increased the uptake of 5-FU by as much as 7-fold in BEL-7402 cells (Fig. 2a). More importantly, 5-FU-mediated ROS production increased more than 14-fold (Fig. 2b, c), which indicated that the mechanical effect might not be the only mechanism here although 5-FU was not a sonosensitizer. Sundaram et al. reported that the key mechanism of ultrasound-enhanced chemotherapy might be due to cavitation-generated ROS production [24]. We therefore hypothesized that the main underlying mechanism of low-intensity US combined with 5-FU could well be the combination of mechanical and cavitation effects.

ROS are a class of strong oxidant, which can regulate many signal transduction pathways by directly reacting with proteins, transcription factors and genes to modulate their functions [25]. Emerging evidence showed that excessive levels of ROS stress might be one of the major causes that triggered the apoptosis of SDT-treated cells [26, 27]. It was reported that 5-FU-mediated ROS contributed to the upregulation of p53 and played a vital role through the induction of apoptosis [17]. Depending on the cellular context and the nature of the DNA damage, 5-FU-mediated p53 had the potential to further trigger the apoptosis signaling pathway through the induction of pro-apoptotic factor-Bax, and the downregulation of anti-apoptotic factor-Bcl-2 [22, 28]. Bax mediated mitochondrial outer membrane permeabilization and regulated cytochrome c release from mitochondria [29]. Subsequently, cytochrome c associated with procaspase-9/Apaf-1 activated effector caspase-3 to initiate the mitochondria-caspase pathway, thus inducing cellular apoptosis [30]. Through the in vitro and in vivo evidence (Fig. 5b, c), we confirmed that the increased ROS enhanced the expressions of p53 protein and found that the protein level of Bcl-2 was significantly down-regulated, while Bax and cleaved caspase-3 protein expressions increased in the US + 5-FU treatment group cells. Compared with the Control, US, and 5-FU groups, the mitochondrial membrane potential in the US + 5-FU group was severely suppressed (Fig. 4a, b) and there was an obvious translocation of cytochrome c from mitochondria into the cytoplasm after the US + 5-FU treatment (Fig. 5a). The elevation of ROS production and the activation of apoptosis-related proteins further motivated the mitochondria-caspase pathway and ultimately led to cell apoptosis. This enhanced cell apoptosis could be partially rescued by the ROS scavenger NAC or pan-caspase inhibitor Z-VAD-FMK (Fig. 6). Thus, we believed that the increased ROS production triggered by the US + 5-FU treatment was the main effector that caused the increased tumor cell apoptosis.

In previous reports, p53 was proven to elevate ROS production by activating ROS-generating proteins [31, 32]. Herein, we verified that ROS could indeed induce p53 expressions. However, it was not clear whether p53 also induced ROS generation and related priorities. Interestingly, p53 was shown to decrease ROS by regulating the expression of antioxidant proteins in some reports [33, 34]. It was also noted that low levels of H_2_O_2_ activated the tumor suppressor p53 to produce an antioxidant response, but high levels of H_2_O_2_ might cause p53-dependent apoptosis [35]. Therefore, we speculated that if the increase of ROS reached a certain threshold level that overwhelmed the antioxidant capacity of cancer cells, ROS might exert a cytotoxic effect, leading to oxidative damage to malignant cells, thus limiting cancer progression [36].

Interestingly, we found that the antioxidant NAC, which was a ROS scavenger, could not completely eliminate ROS production. This implied that there were different kinds of ROS, some of them were not responsive to NAC. Paradoxically, Chinery et al. found that antioxidants pyrrolidinedithiocarbamate and vitamin E enhanced 5-FU-mediated anti-tumor effects in colorectal cancer [37], while the antioxidant NAC had the opposite effects in our study. Possible explanation was that NAC might enhance chemotherapy in p53-dependent manner.

In addition, as described by Yu et al. [38], there was no denying that ultrasound enhanced the action of drugs in most preclinical trials, but the improvement was demonstrated in only limited clinical trials. This implied that the efficacy of ultrasonic chemotherapy could depend on the drugs, cell type, and tumor microenvironment so on. Inappropriate experimental methods could also lead to biases in preclinical trials. Maybe the data in an ectopic model had poor clinical relevancy compared with in an orthotopic model. The use of ultrasonic chemotherapy for clinical application should be improved by optimizing the methods such as the sequence and interval of drug administration or insonation in some cancer types. Anyway, our study could provide feedback for preclinical and translational medicine in ultrasonic chemotherapy.

## Conclusions

In conclusion, we have studied the efficacy and mechanism of US + 5-FU treatment on hepatocellular carcinoma. Our results indicated that the primary mechanism for the much enhanced anticancer effect was via the increased ROS production, which triggered the mitochondria-caspase pathway of apoptosis. It should be noted that low-intensity US combined with 5-FU treated nude mice survived 30 % longer than the untreated Control group and more than 15 % longer than the 5-FU alone group, which was significant. Our results suggested that properly controlled low-intensity US could greatly enhance the localized anticancer effect of 5-FU. In a more general sense, the combined US + Chemotherapy-Drug treatment might offer an innovative way to effectively reduce the drug dosage so as to minimize side effects of conventional chemotherapy.

## Additional files

Additional file 1: Selection of suitable dosages of 5-FU. **Figure S1.**Selection of suitable dosages of 5-FU in vitro and in vivo. Cell viability (a) with 5-FU (0, 2, 5, 10, 20, 40, 60, 80, and 100 μg/ml) and tumor-bearing nude mice (b) in the Control and 5-FU-treated (5, 10, and 20 mg/kg) groups. Data was represented as the mean ± SD (*n* = 6, ^★^ *p* < 0.05 *vs.* Control group). (TIF 793 kb) Additional file 2: Activation of apoptosis-related proteins by low-intensity ultrasound combined with 5-FU. **Figure S2.** Activation of apoptosis-related proteins by low-intensity ultrasound combined with 5-FU in vivo. Protein levels of p53 (a), Bcl-2 (b), Bax (c) and cleaved caspase-3 (d) were evaluated by immunohistochemisty in the Control, US, 5-FU, and US + 5-FU groups. (TIF 8284 kb) Additional file 3: SEM-imgaes of BEL-7402 cells exposed to low-intensity ultrasound. **Figure S3. **SEM-imgaes of BEL-7402 cells exposed to low-intensity ultrasound. The cells were treated without ultrasound (a) and with ultrasound (b). The white arrows pointed out some sonoporation pores. (TIF 1542 kb)

## Abbreviations

5-FU: 5-fluorouracil
H&E staining: hematoxylin and eosin staining
HCC: hepatocellular carcinoma
HPLC: high-performance liquid chromatography
NAC: N-acetylcysteine
PCNA: proliferation cell nuclear antigen
ROS: reactive oxygen species
SDT: sonodynamic therapy
TUNEL: terminal deoxynucleotidyl transferase-mediated dutp nick end-labeling
US: ultrasound
US + 5-FU: low-intensity ultrasound combined with 5-fluorouracil

## References

[1] Allemani C, Weir HK, Carreira H, Harewood R, Spika D, Wang XS (2015). Global surveillance of cancer survival 1995–2009: analysis of individual data for 25 676 887 patients from 279 population-based registries in 67 countries (CONCORD-2). Lancet.

[2] Ferlay J, Soerjomataram I, Dikshit R, Eser S, Mathers C, Rebelo M (2015). Cancer incidence and mortality worldwide: sources, methods and major patterns in GLOBOCAN 2012. Int J Cancer.

[3] Scudellari M (2014). Drug development: try and try again. Nature.

[4] Longley DB, Harkin DP, Johnston PG (2003). 5-fluorouracil: mechanisms of action and clinical strategies. Nat Rev Cancer.

[5] Qin S, Bai Y, Lim HY, Thongprasert S, Chao Y, Fan J (2013). Randomized, multicenter, open-label study of oxaliplatin plus fluorouracil/leucovorin versus doxorubicin as palliative chemotherapy in patients with advanced hepatocellular carcinoma from Asia. J Clin Oncol.

[6] McHale AP, Callan JF, Nomikou N, Fowley C, Callan B (2016). Sonodynamic therapy: concept, mechanism and application to cancer treatment. Adv Exp Med Biol.

[7] Trendowski M (2014). The promise of sonodynamic therapy. Cancer Metastasis Rev.

[8] Yu T, Wang Z, Mason TJ (2004). A review of research into the uses of low level ultrasound in cancer therapy. Ultrason Sonochem.

[9] Tinkov S, Coester C, Serba S, Geis NA, Katus HA, Winter G (2010). New doxorubicin-loaded phospholipid microbubbles for targeted tumor therapy: in-vivo characterization. J Control Release.

[10] Todorova M, Agache V, Mortazavi O, Chen B, Karshafian R, Hynynen K (2013). Antitumor effects of combining metronomic chemotherapy with the antivascular action of ultrasound stimulated microbubbles. Int J Cancer.

[11] Goertz DE, Todorova M, Mortazavi O, Agache V, Chen B, Karshafian R (2012). Antitumor effects of combining docetaxel (taxotere) with the antivascular action of ultrasound stimulated microbubbles. PLoS One.

[12] Yu T, Yang Y, Liu S, Yu H (2009). Ultrasound increases DNA damage attributable to cisplatin in cisplatin-resistant human ovarian cancer cells. Ultrasound Obstet Gynecol.

[13] Li H, Fan H, Wang Z, Zheng J, Cao W (2013). Potentiation of scutellarin on human tongue carcinoma xenograft by low-intensity ultrasound. PLoS One.

[14] He H, Huang H, Yu T (2014). Detection of DNA damage in sonochemotherapy against cisplatin-resistant human ovarian cancer cells using the modified comet assay. Int J Radiat Biol.

[15] Hu Z, Fan H, Lv G, Zhou Q, Yang B, Zheng J (2015). 5-aminolevulinic acid-mediated sonodynamic therapy induces anti-tumor effects in malignant melanoma via p53-miR-34a-Sirt1 axis. J Dermatol Sci.

[16] Fan HX, Li HX, Chen D, Gao ZX, Zheng JH (2012). Changes in the expression of MMP2, MMP9, and ColIV in stromal cells in oral squamous tongue cell carcinoma: relationships and prognostic implications. J Exp Clin Cancer Res.

[17] Hwang PM, Bunz F, Yu J, Rago C, Chan TA, Murphy MP (2001). Ferredoxin reductase affects p53-dependent, 5-fluorouracil-induced apoptosis in colorectal cancer cells. Nat Med.

[18] Lamberti M, Porto S, Marra M, Zappavigna S, Grimaldi A, Feola D (2012). 5-fluorouracil induces apoptosis in rat cardiocytes through intracellular oxidative stress. J Exp Clin Cancer Res.

[19] Wood AK, Sehgal CM (2015). A review of Low-intensity ultrasound for cancer therapy. Ultrasound Med Biol.

[20] Dalecki D (2004). Mechanical bioeffects of ultrasound. Annu Rev Biomed Eng.

[21] Mohamed MM, Mohamed MA, Fikry NM (2003). Enhancement of antitumor effects of 5-fluorouracil combined with ultrasound on Ehrlich ascites tumor in vivo. Ultrasound Med Biol.

[22] Chumakova OV, Liopo AV, Evers BM, Esenaliev RO (2006). Effect of 5-fluorouracil, optison and ultrasound on MCF-7 cell viability. Ultrasound Med Biol.

[23] Lentacker I, De Cock I, Deckers R, De Smedt SC, Moonen CT (2014). Understanding ultrasound induced sonoporation: definitions and underlying mechanisms. Adv Drug Deliv Rev.

[24] Sundaram J, Mellein BR, Mitragotri S (2003). An experimental and theoretical analysis of ultrasound-induced permeabilization of cell membranes. Biophys J.

[25] Trachootham D, Alexandre J, Huang P (2009). Targeting cancer cells by ROS-mediated mechanisms: a radical therapeutic approach?. Nat Rev Drug Discov.

[26] Yumita N, Iwase Y, Nishi K, Komatsu H, Takeda K, Onodera K (2012). Involvement of reactive oxygen species in sonodynamically induced apoptosis using a novel porphyrin derivative. Theranostics.

[27] Yumita N, Okudaira K, Momose Y, Umemura S (2010). Sonodynamically induced apoptosis and active oxygen generation by gallium-porphyrin complex, ATX-70. Cancer Chemother Pharmacol.

[28] Wang JM, Xiao BL, Zheng JW, Chen HB, Zou SQ (2007). Effect of targeted magnetic nanoparticles containing 5-FU on expression of bcl-2, bax and caspase 3 in nude mice with transplanted human liver cancer. World J Gastroenterol.

[29] Borralho PM, da Silva IB M, Aranha MM, Albuquerque C, Nobre Leitao C, Steer CJ (2007). Inhibition of Fas expression by RNAi modulates 5-fluorouracil-induced apoptosis in HCT116 cells expressing wild-type p53. Biochim Biophys Acta.

[30] Adachi Y, Taketani S, Oyaizu H, Ikebukuro K, Tokunaga R, Ikehara S (1999). Apoptosis of colorectal adenocarcinoma induced by 5-FU and/or IFN-gamma through caspase 3 and caspase 8. Int J Oncol.

[31] Donald SP, Sun XY, Hu CA, Yu J, Mei JM, Valle D (2001). Proline oxidase, encoded by p53-induced gene-6, catalyzes the generation of proline-dependent reactive oxygen species. Cancer Res.

[32] Rivera A, Maxwell SA (2005). The p53-induced gene-6 (proline oxidase) mediates apoptosis through a calcineurin-dependent pathway. J Biol Chem.

[33] Bensaad K, Tsuruta A, Selak MA, Vidal MN, Nakano K, Bartrons R (2006). TIGAR, a p53-inducible regulator of glycolysis and apoptosis. Cell.

[34] Sablina AA, Budanov AV, Ilyinskaya GV, Agapova LS, Kravchenko JE, Chumakov PM (2005). The antioxidant function of the p53 tumor suppressor. Nat Med.

[35] D'Autreaux B, Toledano MB (2007). ROS as signalling molecules: mechanisms that generate specificity in ROS homeostasis. Nat Rev Mol Cell Bio.

[36] Chen G, Wang F, Trachootham D, Huang P (2010). Preferential killing of cancer cells with mitochondrial dysfunction by natural compounds. Mitochondrion.

[37] Chinery R, Brockman JA, Peeler MO, Shyr Y, Beauchamp RD, Coffey RJ (1997). Antioxidants enhance the cytotoxicity of chemotherapeutic agents in colorectal cancer: a p53-independent induction of p21WAF1/CIP1 via C/EBPbeta. Nat Med.

[38] Yu T, Luo L, Wang L (2016). Ultrasound as a cancer chemotherapy sensitizer: the gap between laboratory and bedside. Expert Opin Drug Deliv.

