# Supplementary figures and images for: Enhancement of anti-tumor effects of 5-fluorouracil on hepatocellular carcinoma by low-intensity ultrasound
Source: J Exp Clin Cancer Res. 2016 Apr 22;35:71. doi: 10.1186/s13046-016-0349-4 (PMC4840943; doi:10.1186/s13046-016-0349-4)

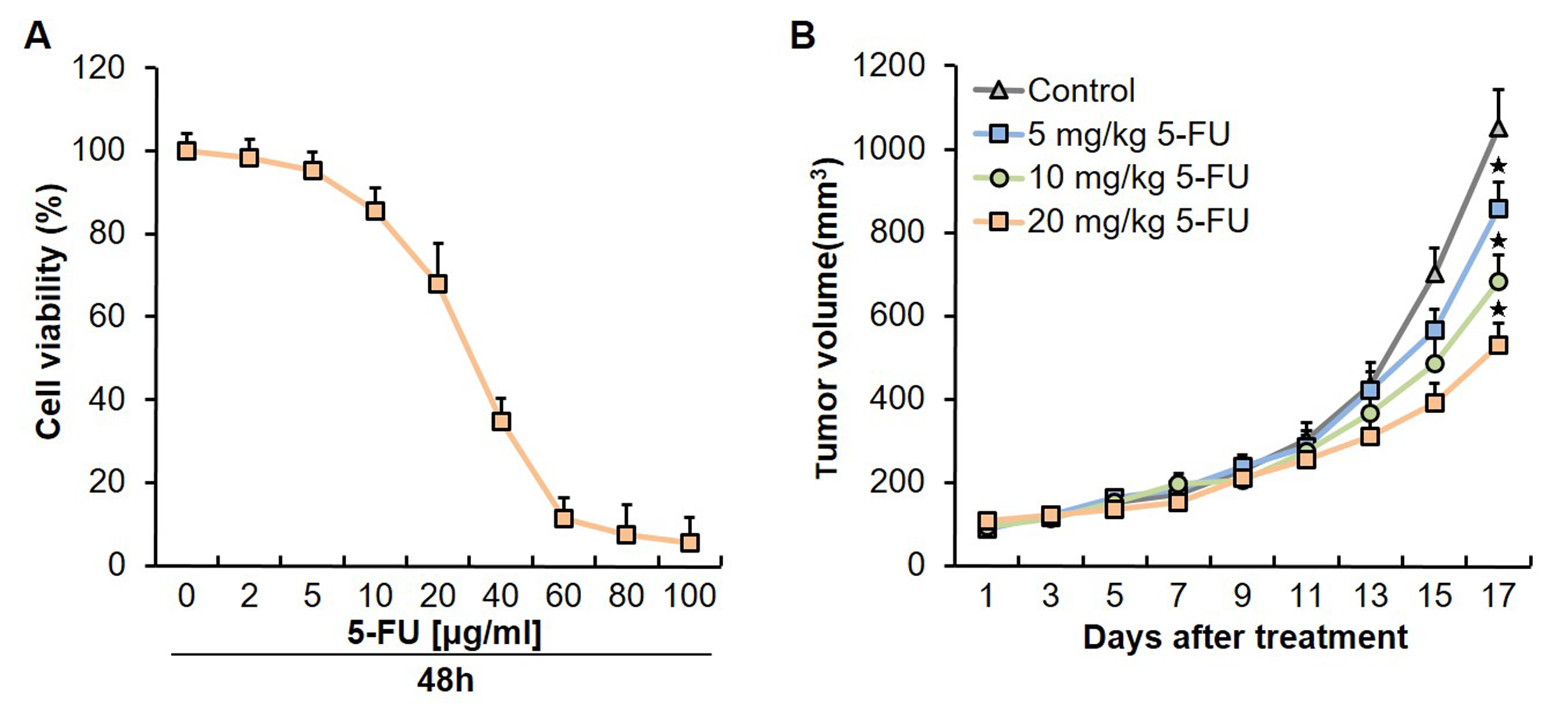

Supplement: Additional file 1: — Selection of suitable dosages of 5-FU. Figure S1.Selection of suitable dosages of 5-FU in vitro and in vivo. Cell viability (a) with 5-FU (0, 2, 5, 10, 20, 40, 60, 80, and 100 μg/ml) and tumor-bearing nude mice (b) in the Control and 5-FU-treated (5, 10, and 20 mg/kg) groups. Data was represented as the mean ± SD (n = 6, ★ p < 0.05 vs. Control group). (TIF 793 kb) [file 13046_2016_349_MOESM1_ESM.tif]

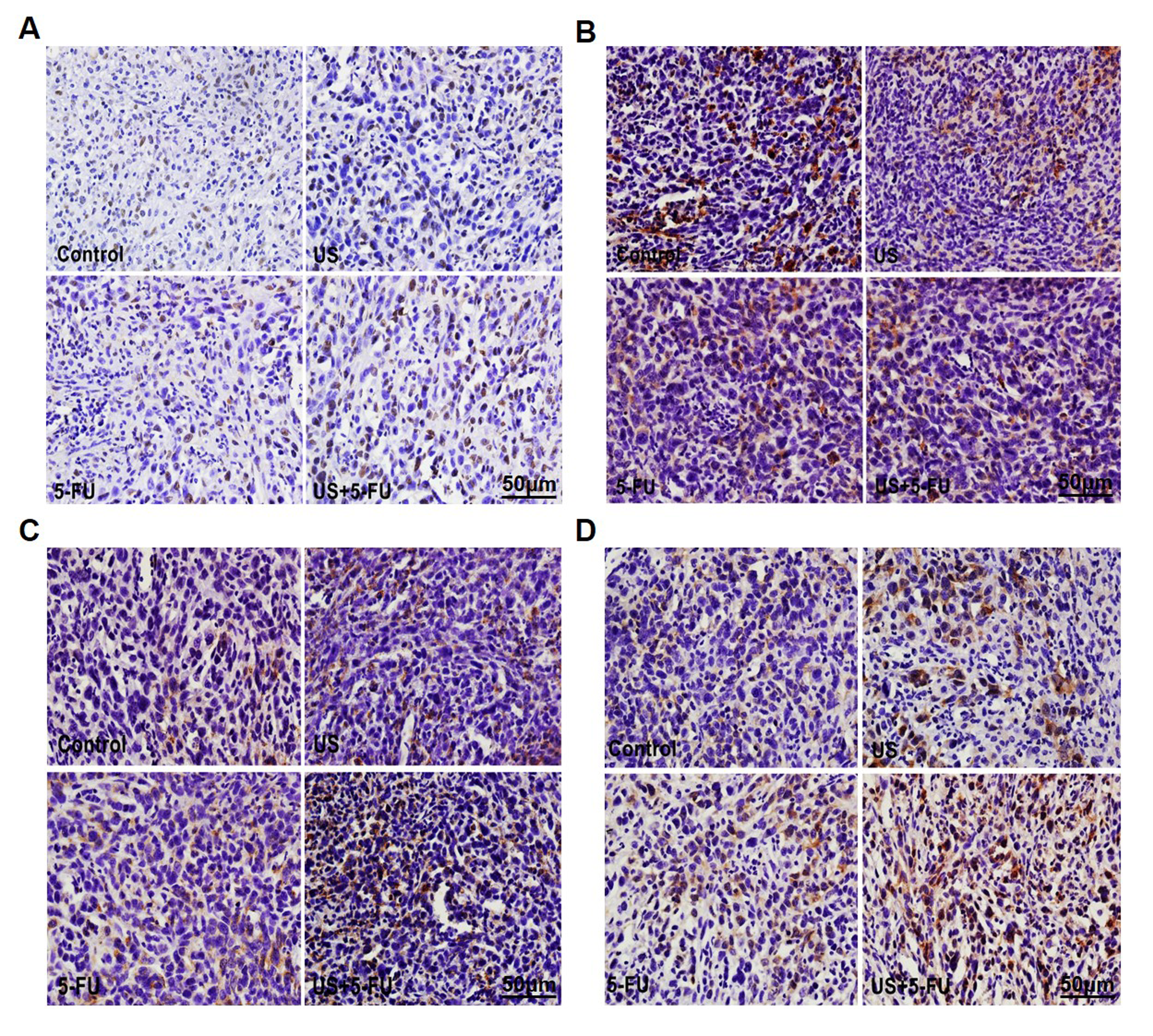

Supplement: Additional file 2: — Activation of apoptosis-related proteins by low-intensity ultrasound combined with 5-FU. Figure S2. Activation of apoptosis-related proteins by low-intensity ultrasound combined with 5-FU in vivo. Protein levels of p53 (a), Bcl-2 (b), Bax (c) and cleaved caspase-3 (d) were evaluated by immunohistochemisty in the Control, US, 5-FU, and US + 5-FU groups. (TIF 8284 kb) [file 13046_2016_349_MOESM2_ESM.tif]

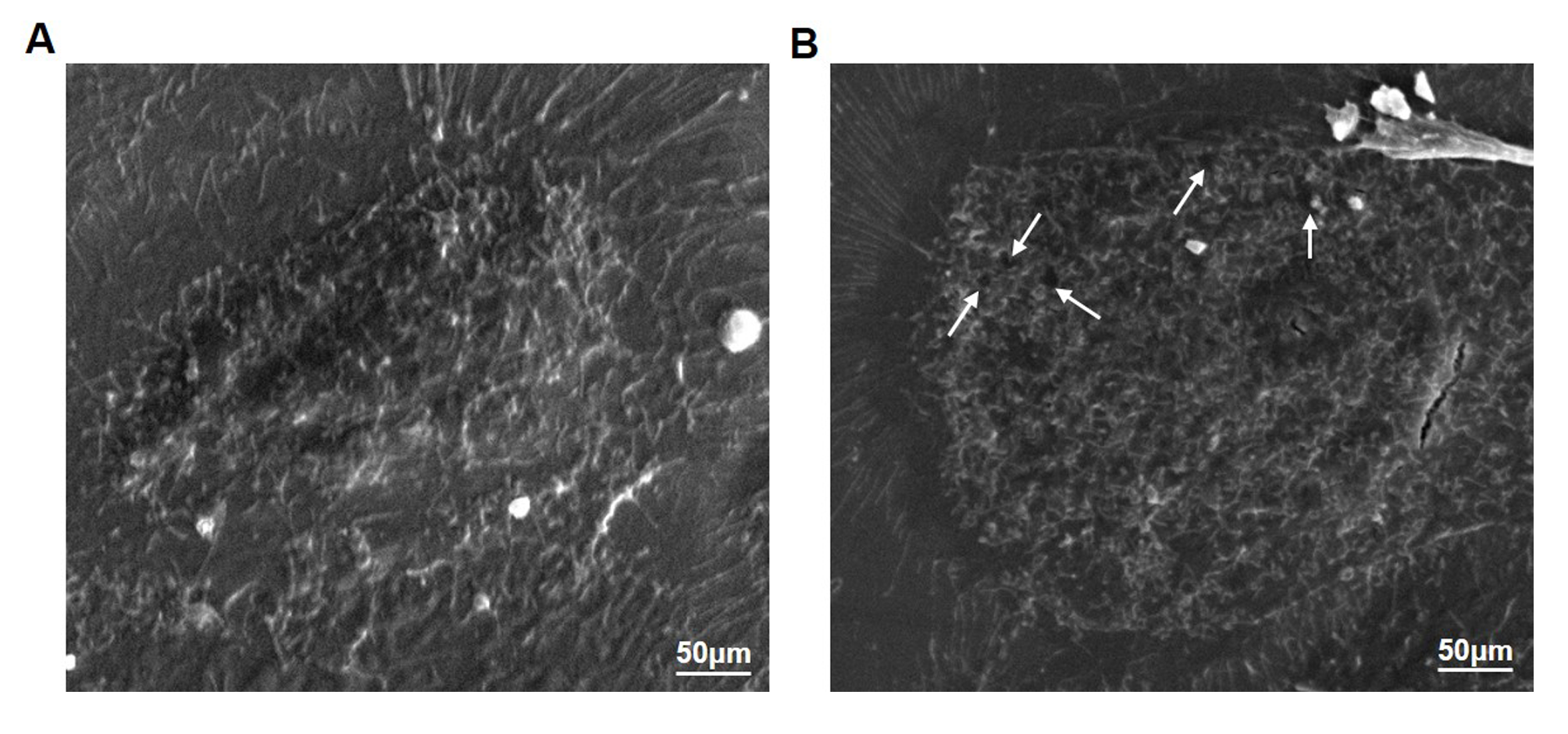

Supplement: Additional file 3: — SEM-imgaes of BEL-7402 cells exposed to low-intensity ultrasound. Figure S3. SEM-imgaes of BEL-7402 cells exposed to low-intensity ultrasound. The cells were treated without ultrasound (a) and with ultrasound (b). The white arrows pointed out some sonoporation pores. (TIF 1542 kb) [file 13046_2016_349_MOESM3_ESM.tif]
